# Central mechanisms mediating the hypophagic effects of oleoylethanolamide and *N*-acylphosphatidylethanolamines: different lipid signals?

**DOI:** 10.3389/fphar.2015.00137

**Published:** 2015-06-26

**Authors:** Adele Romano, Bianca Tempesta, Gustavo Provensi, Maria B. Passani, Silvana Gaetani

**Affiliations:** ^1^Department of Physiology and Pharmacology “V. Erspamer”, Sapienza University of Rome, Rome,Italy; ^2^Department of Neuroscience, Psychology, Drug Discovery and Child Health (NEUROFARBA), University of Florence, Florence, Italy

**Keywords:** oleoylethanolamide, *N*-acylphosphatidylethanolamines, oxytocin, histamine, hypothalamus, nucleus of the solitary tract, satiety and food intake, obesity

## Abstract

The spread of “obesity epidemic” and the poor efficacy of many anti-obesity therapies in the long-term highlight the need to develop novel efficacious therapy. This necessity stimulates a large research effort to find novel mechanisms controlling feeding and energy balance. Among these mechanisms a great deal of attention has been attracted by a family of phospholipid-derived signaling molecules that play an important role in the regulation of food-intake. They include *N*-acylethanolamines (NAEs) and *N*-acylphosphatidylethanolamines (NAPEs). NAPEs have been considered for a long time simply as phospholipid precursors of the lipid mediator NAEs, but increasing body of evidence suggest a role in many physiological processes including the regulation of feeding behavior. Several observations demonstrated that among NAEs, oleoylethanolamide (OEA) acts as a satiety signal, which is generated in the intestine, upon the ingestion of fat, and signals to the central nervous system. At this level different neuronal pathways, including oxytocinergic, noradrenergic, and histaminergic neurons, seem to mediate its hypophagic action. Similarly to NAEs, NAPE (with particular reference to the N16:0 species) levels were shown to be regulated by the fed state and this finding was initially interpreted as fluctuations of NAE precursors. However, the observation that exogenously administered NAPEs are able to inhibit food intake, not only in normal rats and mice but also in mice lacking the enzyme that converts NAPEs into NAEs, supported the hypothesis of a role of NAPE in the regulation of feeding behavior. Indirect observations suggest that the hypophagic action of NAPEs might involve central mechanisms, although the molecular target remains unknown. The present paper reviews the role that OEA and NAPEs play in the mechanisms that control food intake, further supporting this group of phospholipids as optimal candidate for the development of novel anti-obesity treatments.

## Oleoylethanolamide and NAPE as Lipid Mediators

The growing prevalence of obesity and the limited pharmacological therapies currently available to control overfeeding of obese patients in an efficacious and safe manner highlights the need to identify novel neuro-molecular mechanisms involved in the control of food intake for the development of new drugs. This research area has expanded rapidly and led to the description of a large multitude of neuroanatomical, neurochemical, and genetic mechanisms (Benoit et al., 2008; Figlewicz and Sipols, 2010) organized in a complex, redundant, and highly integrated network of several endogenous molecules of heterogeneous nature, such as peptides, hormones, and lipids (Matias et al., 2006; Diéguez et al., 2011; Ghourab et al., 2011; Lateef et al., 2011; Parker and Bloom, 2012; Tsuneki et al., 2012). Within the lipid compounds a great deal of attention has been recently dedicated to a family of phospholipid-derived signaling molecules identified to play an important role in the regulation of food-intake. They include *N*-acylethanolamines (NAEs; Rodríguez de Fonseca et al., 2001; Fu et al., 2007; Dipasquale et al., 2011; Piomelli, 2013; D’Addario et al., 2014) and *N*-acylphosphatidylethanolamines (NAPEs; Gillum et al., 2008).

**FIGURE 1:**
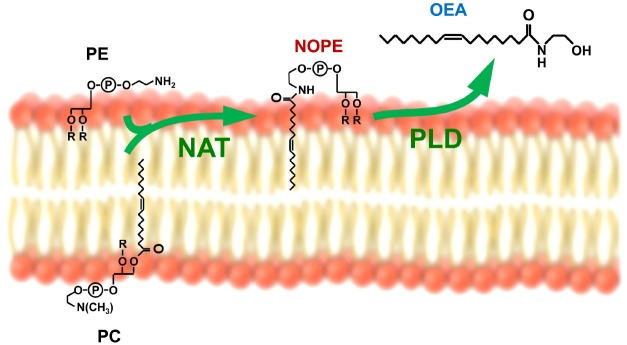
**The synthesis of oleoylethanolamide (OEA) is mediated by two concerted reactions: The first one is the *N*-acylation of phosphatidylethanolamine (PE) from the phospholipid bilayer of the cell membrane, mediated by a N-acyltransferase (NAT) to form *N*-oleoylphosphatidylethanolamine (NOPE) and the second one is the phospholipase D (PLD)-mediated hydrolysis of NOPE.** Similar enzymatic reactions take place also during the synthesis of other NAEs, so that NAPEs are synthesized by acylation of the free amine group of ethanolamine-containing glycerophospholipids (both those containing diacyl and those containing alkenyl-acyl bound fatty acids) and transformed into the respective NAEs by NAPE-dependent PLD.

*N*-acylethanolamines have been the most widely characterized. They include anandamide that was the most studied among them for its ability to act as endogenous cannabinoid ligand (D’Addario et al., 2014). Besides cannabinoid receptors, it has been gradually demonstrated that, depending on the nature of the *N*-linked acyl chain, NAEs can interact with different receptors in animals, including also vanilloid receptors and peroxisome proliferator activated-receptors (PPAR), and to be involved in many physiological processes (Fu et al., 2003; Nielsen et al., 2004; Terrazzino et al., 2004; Diep et al., 2011). With regard to the role played in the regulation of food intake, the most studied NAE was the monounsaturated analog of anandamide, oleoylethanolamide (OEA). Produced primarily in the small intestine, upon the absorption of dietary fat, OEA was demonstrated to act as a satiety signal in rodents by prolonging the interval between meals (Fu et al., 2003; Gaetani et al., 2003; Karimian Azari et al., 2014; Romano et al., 2014a). OEA endogenous mobilization is under the control of the sympathetic nervous system (LoVerme et al., 2006a; Fu et al., 2011) and is mediated by two concerted reactions. The first one is the transfer of an oleic group from the stereospecific numbering-1 (sn-1) position of a membrane phospholipid (for example phosphatidylcholine) to the amine group of a second membrane phospholipid, namely phosphatidylethanolamine (PE; Figure 1). This reaction is catalyzed by an enzyme, which remains to be molecularly identified, and whose N-acyltransferase activity seems to be calcium-dependent. The products of this reaction form a chemically heterogeneous family of *N*-oleoylphosphatidylethanolamines (NOPEs), which, depending on the specific PE precursor involved, may differ in the substituents at sn-1 and sn-2 position of PE (Figure 1). The sn-1 position is most often esterified by a saturated fatty acid or can contain an alkyl or alkenyl ether moiety; while the sn-2 position usually binds an unsaturated or polyunsaturated fatty acid (Coulon et al., 2012). Similar enzymatic reactions take place also during the synthesis of other NAEs, so that NAPEs are synthesized by acylation of the free amine group of ethanolamine-containing glycerophospholipids (both those containing diacyl and those containing alkenyl-acyl bound fatty acids; Natarajan et al., 1982; Schmid et al., 1990). This mechanism for NAEs formation was described some time ago and suggested that NAPEs merely act as unstable intermediate metabolites quickly converted into NAEs. However, novel evidence suggested that rather than simply precursors to NAEs, NAPEs can be defined as signaling lipids, able to control important biological functions on their own (Mora et al., 2002; Wellner et al., 2013). For example, it was recently demonstrated that among NAPEs, N-palmitoyl-phosphatidylethanolamine (N16:0 NAPE) can be involved in the gut-to brain axis that regulates food intake (Gillum et al., 2008; Fu et al., 2011).

The second reaction is catalyzed by a NAPE-selective phospholipase D (NAPE-PLD; Figure 1), which was purified and molecularly cloned by Dr. Ueda and his collaborators (Okamoto et al., 2004). NAPE-PLD hydrolyses different analogs of the NAPE family with similar efficiency. Targeted deletion of the NAPE-PLD gene (Napepld) in mice did not completely prevent OEA endogenous synthesis, thus suggesting the existence of redundant biosynthetic pathways (Simon and Cravatt, 2010; Tsuboi et al., 2011).

## OEA and the Control of Food Intake

The natural lipid mediator OEA contributes to the peripheral regulation of feeding by evoking a consistent and sustained inhibition of food intake in rats and mice, after both intraperitoneal (i.p.) and oral administration (Rodríguez de Fonseca et al., 2001; Fu et al., 2003; Gaetani et al., 2003; Nielsen et al., 2004; Oveisi et al., 2004; Proulx et al., 2005; Astarita et al., 2006; Karimian Azari et al., 2014; Romano et al., 2014a).

Rats start their feeding activity around dark onset, with a variable latency. The activity is organized into episodes called “meals” that can correspond to a variable amount of food eaten (meal size). Meals are separated by intervals of variable duration (inter-meal interval; Gaetani et al., 2003; Karimian Azari et al., 2014; Romano et al., 2014a). Anorexic agents and feeding-regulating hormones act by modifying different aspects of this patterned behavior. The effect of OEA differs mechanistically from those of the serotonergic anorexiant *d*-fenfluramine, which affects both latency and meal size (Clifton et al., 2000), and of the intestinal peptide CCK, which mostly reduces meal size (Ritter et al., 1999). In particular, the anorexiant effect of OEA depends on the feeding state of the rats (Gaetani et al., 2003; Karimian Azari et al., 2014). In fact i.p. administration of OEA (1–20 mg/kg) causes a dose-dependent delay in the feeding onset in free feeding animals without altering the behavioral pattern of feeding, whereas it evokes both delayed feeding onset and reduced meal size in food-deprived rats (Gaetani et al., 2003; Karimian Azari et al., 2014). On virtue of the variety of different factors that can influence feeding in a non-specific manner, it is important to underlie that OEA suppresses feeding without causing stress, visceral illness and without affecting locomotor activity (Rodríguez de Fonseca et al., 2001; Proulx et al., 2005). Moreover the behavioral specificity of the anorexigenic effect of OEA is further supported by the observation that OEA does not affect drinking behavior or sodium appetite (Rodríguez de Fonseca et al., 2001; Proulx et al., 2005). The anorexiant effect of OEA is also structurally selective, as the endocannabinoid anandamide, and close analogs of OEA, such as oleic acid, linoleoylethanolamide (LEA), and palmitoylethanolamide (PEA) are inactive or less active than OEA, when administered i.p. to laboratory rats (Rodríguez de Fonseca et al., 2001; Hansen and Diep, 2009; Diep et al., 2011). In particular, PEA is significantly less potent than OEA in reducing food intake; LEA is similar in potency to OEA and anandamide and oleic acid have no effect (Rodríguez de Fonseca et al., 2001; Diep et al., 2011).

The endogenous levels of OEA, as well as of PEA and LEA, are regulated by the nutritional status of the animals. In fact, fasting decreases NAEs levels in the first tract of the small intestine, while food ingestion stimulates cells in the mucosal layer of the duodenum and jejunum to produce endogenous OEA (Fu et al., 2007, 2011), PEA and LEA (Diep et al., 2011; Igarashi et al., 2015). This observation further supports the hypothesis that endogenous OEA might participate to the induction of satiety (Dipasquale et al., 2011; Piomelli, 2013). In support of this idea, the enhancement of food-stimulated mobilization of OEA in the intestine, obtained by a viral-vector-mediated overexpression of NAPE-PLD into the duodenum of rats, elicits a satiety effect behaviorally similar to that observed after the systemic administration of exogenous OEA (Fu et al., 2008). This evidence further points to the role of endogenous OEA in the small intestine as a local satiety messenger.

Moreover, the observation that the endogenous levels of intestinal OEA, LEA and PEA can be affected by the fat content of the diet in a time- and dose-dependent manner, suggested a possible role of these anorexiant NAEs as intestinal fat sensor (Diep et al., 2011). This hypothesis is further supported by the recent finding that such mechanism is altered in animals rendered obese by the exposure to a high fat diet or in animals fed with a high-sucrose low-fat diet, in which OEA and LEA mobilizations from the intestine do not respond to the intraduodenal infusion of lipids (Igarashi et al., 2015). These observations suggest that alterations of the anorexiant NAE pathways might contribute to hyperphagia and to the development of obesity.

## Peroxisome Proliferator-activated Receptor (PPAR)-alpha Mediates the Satiety-inducing Effects of OEA

Several lines of evidences indicate that OEA is a high-affinity agonist of the nuclear PPAR-alpha, the molecular target of the antihyperlipidemic drugs fibrates, whereas it does not engage PPAR-gamma or retinoid-X receptor—a functional partner in PPAR-activated transcription (Fu et al., 2003). OEA is a more potent activator of PPAR-alpha (EC_50_ = 120 nM) compared to other natural ligands, such as oleic acid (EC_50_ = 10 μM) and synthetic agonists, such as GW7647 (EC_50_ = 150 nM) (Fu et al., 2003; Lo Verme et al., 2005a). Saturation binding experiments show that OEA binds to the purified ligand-binding (LBD) domain of PPAR-alpha with a *K*_D_ of approximately 40 nM (Fu et al., 2003; Lo Verme et al., 2005b). Furthermore, experiments in genetically modified mice show that OEA does not reduce feeding in animals lacking a functional PPAR-alpha gene, suggesting that PPAR-alpha activation crucially contributes to the hypophagic actions of OEA (Fu et al., 2003; Lo Verme et al., 2005b). In support of the selectivity of such effect, PPAR-alpha deficient mice retain the ability to respond to other anorexiant agent, such as *d*-fenfluramine and cholecystokinin (Fu et al., 2003). Moreover PPAR-alpha deficient mice show decreased eating latency and increased feeding frequency, without other alterations of ingestive behavior (Schwartz et al., 2008). The possibility that OEA evokes hypophagia by activating PPAR-alpha is further supported by several findings: (i) the two potent synthetic PPAR-alpha agonists GW7647 and Wy-14643 inhibit food intake, while fibrates, which are weak PPAR-alpha agonists (Kersten and Wahli, 2000), have no anorexiant effect; (ii) the administration of OEA or synthetic PPAR-alpha agonists alter the expression of PPAR-alpha -regulated genes in the small intestine and liver (Fu et al., 2003); (iii) food intake increases OEA concentrations in the intestinal mucosa to levels (∼300 nM) that are sufficient to fully activate PPAR-alpha (Fu et al., 2007, 2011). Moreover, PPAR-alpha activation induces several transcriptional changes that lead to an increase of fatty-acid catabolism, a reduction of blood lipid levels and a decrease of body-weight gain (Willson et al., 2000; Berger and Moller, 2002): the chronic treatment with OEA in rodent models of obesity can produce all these effects (Rodríguez de Fonseca et al., 2001; Fu et al., 2005).

Besides PPAR*-*alpha receptor, other molecular targets have been proposed to mediate OEA’s actions *in vivo* (Dipasquale et al., 2011; Piomelli, 2013). These include the transient receptor potential cation channel vanilloid-1 (TRPV1) and the “orphan” G-coupling receptor, GPR119. The evidence supporting the interaction with the TRPV1 came from few studies. The first one was performed on Xenopus oocytes and showed that OEA can activate this receptor (with an EC_50_ of approximately 2 μM) once it has been phosphorylated by protein kinase C (PKC; Ahern, 2003). The second study demonstrated that OEA is able to depolarize capsaicin-sensitive nodose ganglion neurons cultured from wild type (wt) mice by evoking inward currents that can be blocked by the TRPV1 inhibitor, capsazepine, while no response to OEA was observed in neurons cultured from TRPV1-null mice (Wang et al., 2005). In the same study, authors showed that OEA administration induced visceral pain-related behaviors in wt mice, but not in TRPV1-null animals or in wt mice pre-treated with capsazepine (Wang et al., 2005). Similar observations were reported also by LoVerme et al. (2006b) showing that intraplantar administration of OEA (50 μg) into the hind paw can elicit nocifensive behavior in wt, but not in TRPV1-null mice.

Moreover, OEA was suggested to act also as a medium-potency (EC_50_ ∼3 μM) endogenous ligand of the orphan receptor GPR119, a G protein-coupled receptor expressed predominantly in the human and rodent pancreas and gastrointestinal tract and also in rodent brain (Overton et al., 2006). Through the activation of this receptor, OEA was shown to increases GLP-1 secretion from intestinal L-cells (Lauffer et al., 2009).

However, despite these observations on OEA actions at other possible pharmacological targets different from PPAR-alpha, it is noteworthy that the genetic deletion of either TRPV1 or GPR119 in mice does not prevent the anorexigenic effects of OEA, thus clearly indicating that the activation of these two receptors induced by OEA is not crucial for its action as a satiety signal (Lo Verme et al., 2005b; Lan et al., 2009).

## NAPE and the Control of Food Intake

*N*-acylphosphatidylethanolamines were observed in several tissues and organs, including the nervous system, the gastrointestinal tract, testis, spleen, plasma, and lymph (for review, see Coulon et al., 2012). Within the rat nervous system they are particularly abundant in the spinal cord (Yang et al., 1999) and the brain stem (Bisogno et al., 1999), with a N-acyl moiety that mostly includes N16:0, N18:0, N18:1 (69.6 17%, 12.2 3.3%, 8.1 2.5% respectively) with only a 0.2% of N20:4 (Sugiura et al., 1996).

*N*-acylphosphatidylethanolamine intestinal, plasma and lymph levels can be significantly affected by the fed state of the animals. In fact, most NAPE species (N16:0, N 18:0, N18:1, N18:2) resulted significantly reduced in the intestines of fasted rats and increased within few hours upon re-feeding; the *N* 20:4 NAPE is the only species showing an opposite trend (Petersen et al., 2006; Fu et al., 2007). Such fluctuations were initially linked to the regulation of NAE synthesis (Hansen et al., 2000). However, in 2008 Gillum et al. (2008) proposed, for the first time, that NAPE *per se* can act as a phospholipid hormone involved in the regulation of food intake. In particular, after confirming the previous findings showing that in fasted rats fed with a high fat diet or subjected to intraduodenal lipid infusion NAPE levels increased in plasma, lymph and small intestine, they demonstrated that the exogenous administration of the most abundant plasma NAPE (N 16:0) decreased food intake in rats and mice after both i.p. and intracerebroventricular (i.c.v.) administration, without causing conditioned taste aversion (Gillum et al., 2008).

It is worth noting that N 16:0 NAPE tested in Gillum’s experiment is not OEA precursor. The molecular target of NAPE that is mediating its effect on food intake remains to be discovered. It likely does not involve cannabinoid CB_1_ receptor, as exogenous NAPE was still effective in suppressing food intake in CB_1_ knockout mice (Gillum et al., 2008). Moreover, as demonstrated by another research group, the anorectic effects of NAPE does not seem to be mediated by a NAPE metabolite, such as NAE, as suggested by the observation that the hypophagic action of NAPE can be still observed in mice lacking NAPE-PLD that are unable to convert NAPE into NAE (Wellner et al., 2011).

However, NAPE i.p. administered can exerts its effect at large dosage (100–200 mg/kg), and several other phospholipids have an anorectic effect in the same dose range, when administered i.p. (Wellner et al., 2011). This aspect raised doubts about the specificity of NAPE anorexiant effect (Gillum et al., 2008; Wellner et al., 2011) and questioned the suitability of the i.p. route of administration for a large dose of this compound. Future studies should clarify whether NAPEs and NAEs can act as independent, possibly complementary lipid signals involved in the regulation of food intake.

## Effects Produced by OEA in the Central Nervous System

**FIGURE 2:**
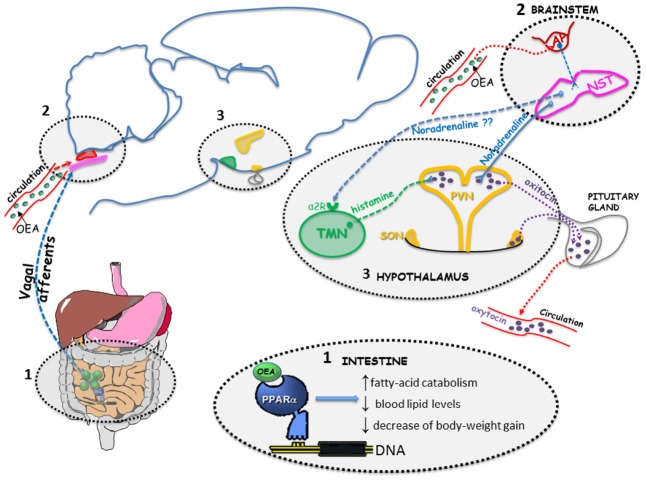
**OEA activates PPAR-alpha receptor in the proximal small intestine (1) generating an input that induces several transcriptional changes leading to an increase of fatty-acid catabolism, a reduction of blood lipid levels and a decrease of body-weight gain.** The signal travels (through a mechanism that still needs to be elucidated) to the brainstem (2), in the nucleus of solitary tract (NST). Circulating OEA might reach the area postrema (AP; a circumventricular organ that lacks a functional blood brain barrier and is in strict contact with the NST). From the noradrenergic neurons within the NST the signal reaches the oxytocinergic neurons of the paraventricular nucleus (PVN) and supraoptic nucleus (SON), where it stimulates oxytocin expression and release, presumably the tuberomamillary nucleus (TMN) as well, where it stimulates histamine release from neurons projecting to the PVN (3). Oxytocin released from neurons of PVN and SON can act centrally to modulate feeding behavior and can be released into the blood stream from the neurohypophysis.

Similarly to other anorexigenic gut-derived signals, peripherally administered OEA (10 mg/kg i.p.) activates c-fos transcription (investigated as marker of neuronal activation) in the nucleus of solitary tract (NST; Rodríguez de Fonseca et al., 2001; Gaetani et al., 2010; Romano et al., 2013, 2014b), and the paraventricular (PVN) and supraoptic (SON) nuclei of the hypothalamus (Rodríguez de Fonseca et al., 2001; Gaetani et al., 2010; Romano et al., 2013, 2014b). In both hypothalamic nuclei, OEA increases c-fos mRNA and protein expression in oxytocin-immunoreactive neurons (Gaetani et al., 2010; Provensi et al., 2014). This activation is paralleled by increased oxytocin mRNA levels, increased peptide neurosecretion, and elevated circulating oxytocin levels (Gaetani et al., 2010; Romano et al., 2013). Moreover, pharmacological blockade of oxytocin receptors in the brain by i.c.v. infusion of the selective oxytocin antagonist, L-368,899, prevented the anorexic effects of OEA, suggesting that OEA suppresses feeding by activating central oxytocinergic transmission (Figure 2; Gaetani et al., 2010).

We recently reported that OEA requires the integrity of the brain histaminergic system to fully exert its hypophagic effect (Provensi et al., 2014). Brain histamine has long been known as a mediator of satiety through activation of hypothalamic histamine H_1_ receptors (reviewed, in Passani et al., 2011). We found that in mice deficient of the histamine synthesizing enzyme histidine decarboxylase (HDC), or acutely depleted of histamine by i.c.v. infusions of the HDC blocker a-fluoromethylhistidine, the effect of exogenously administered OEA was significantly attenuated. Furthermore, OEA increased c-fos expression in oxytocin neurons of the PVN of wt, but not HDC-KO mice, suggesting that oxytocin rich nuclei are the likely brain region where histamine influences the central, indirect effects of OEA. In addition, i.p. injections of OEA increased cortical release of histamine as measured by *in vivo*-microdialysis in fasted mice within a time frame that is compatible with its anorexic effect (Provensi et al., 2014). This observation further supports the hypothesis that histamine signaling is involved in the acute effects of OEA on food consumption (Figure 2). Moreover, in a recent report (Romano et al., 2014b) we showed that i.p. administration of OEA, at a dose and at a time-point that causes a significant inhibition of eating, stimulates c-fos transcription in specific subnuclei of the brainstem. In particular, OEA evoked a strong signal in the area postrema (AP), a circumventricular organ that lacks a functional blood brain barrier, and in the medial and central part of the NST. Interestingly, within the central part of the NST, the induction of c-fos mRNA was more evident at the most rostral level where this nucleus is more in contact with the AP (Romano et al., 2014b). This observation might suggest a direct action of OEA in the brain stem by reaching circumventricular organs from the blood stream (Figure 2).

Noradrenergic projections from the NST to the hypothalamus seem to mediate OEA effects on feeding behavior and on hypothalamic oxytocin increase (Figure 2), as demonstrated in rats subjected to the intra-PVN administration of the toxin saporin (DSAP) conjugated to an antibody against dopamine-β-hydroxylase (DBH) to destroy hindbrain noradrenergic neurons (Romano et al., 2013). In fact, in these rats the lesion induced by DSAP administration completely prevented OEA effect on food intake and on both c-fos and oxytocin expression in the PVN, thus supporting the hypothesis of a necessary role played by the NST-PVN noradrenergic pathway (Romano et al., 2013). In accordance with this observation, peripheral administration of OEA (5–20 mg/kg) to rats increased noradrenaline concentrations in the hypothalamus in a dose-dependent manner (Serrano et al., 2011). In the same study authors demonstrated also that peripherally administered OEA was able to increase hypothalamic dopamine concentrations, but failed to affect the levels of two orexigenic neuropeptides expressed by the arcuate nucleus, such as neuropeptide Y (NPY) or Agouti related-peptide (AgRP), in both 24-h food derived and free-feeding rats (Serrano et al., 2011). A positive effect of OEA was, instead, observed on the levels of the anorexigenic peptide Cocaine and Amphetamine Regulated Transcript (CART) in the PVN but not the arcuate nucleus (Serrano et al., 2011).

The observation that OEA is able to affect monoaminergic transmission in the brain was also reported by other groups. For example, Yu et al. (2015) detected changes in cerebral noradrenaline and serotonin levels following OEA repeated oral administration and linked such effect to the antidepressant-like effect produced in the same animals. Murillo-Rodríguez et al. (2011) locally administered OEA into the lateral hypothalamus or the dorsal raphe of rats and observed, by brain microdialysis of the nucleus accumbens, an increase of dopamine extracellular levels. A modulation of dopaminergic function was reported also by other authors who demonstrated the ability of OEA to modulate nicotinic receptors containing β2 subunits (indicated as β2*-nAChRs) expressed by dopamine neurons that play a key role in the reward system (Melis et al., 2013). Both observations might suggest a role of OEA in the modulation of reward functions. Such hypothesis was confirmed by Tellez et al. (2013), who used OEA subchronic treatments to re-establish a normally functioning reward system in rats that were chronically exposed to a high fat diet. Altogether these reports suggest that the modulation of food intake induced by OEA might not only affect homeostatic systems that control hunger and satiety in the brain, but also, hedonic and non-homeostatic aspect related to the salience of food-related stimuli.

The vagus nerve has been shown to mediate the anorexiant effect of NAE-related compounds (Rodríguez de Fonseca et al., 2001). Total subdiaphragmatic vagotomy (TVX), as well as capsaicin pre-treatment prevented the satiety effect of OEA, which prompted the interpretation that OEA’s effect on eating is mediated by vagal sensory fibers (Rodríguez de Fonseca et al., 2001; Fu et al., 2003). Recently, by using a more specific subdiaphragmatic vagal de-afferentiation (SDA), a surgery that eliminates all abdominal vagal afferents sparing approximately half of the efferents (Norgren and Smith, 1994; Walls et al., 1995), we have demonstrated that OEA did not require intact abdominal vagal afferents to reduce food intake (Karimian Azari et al., 2014).

## Effects Produced by NAPE in the Central Nervous System

The initial evidence that NAPEs’ biological functions might include also actions in the central nervous system (CNS) came from *in vitro* studies on the cytoprotective role of NAPE in different models of neurotoxicity or neurodegeneration (for review, see Coulon et al., 2012). NAPE levels in the brain (as well as in other tissues) increase strongly in response to stress conditions that cause a dramatic rise of intracellular calcium concentration. This phenomenon has been linked to the calcium-dependent acyl-transferase activity of the enzymatic reaction that leads to the synthesis of NAPEs from membrane phospholipid precursors (Coulon et al., 2012).

Whether similar mechanisms are involved in the effects of NAPE on food intake remained unexplored. That the hypophagic actions of NAPEs might be mediated by the direct interaction with CNS targets was suggested by different observations made by Gillum et al. (2008). In particular, they found that the surgical elimination of nervous afferents to the brain from the gastrointestinal tract, obtained by subdiaphragmatic vagotomy, did not block the anorectic effects of NAPE; (i) NAPE exerted its anorexiant effect after both central and systemic administration; (ii) intravenously administered ^14^C-labeled C16:0 NAPE entered the brain, and preferentially accumulated in the hypothalamus; (iii) NAPE administration significantly increased c-fos expression in the PVN and SON and decreased c-fos expression in NPY neurons of the arcuate nucleus, with respect to fasted animals that did not receive the drug. These latter effects strongly resemble those produced by OEA. Therefore, the possibility that they might be induced by a NAPE metabolite rather than NAPE *per se* cannot be excluded.

*N*-acylphosphatidylethanolamine effects are not mediated by other signals affecting food intake, such as leptin or melanocortins. In fact, both ob/ob mice and mice lacking the melanocortin four receptor exhibited a full anorexigenic response to the systemic administration of NAPE and even trended toward enhanced sensitivity (Gillum et al., 2008; Srisai et al., 2011).

Despite these initial observations future studies are necessary to unveil the central target of NAPEs and to further explore their biological role in the CNS.

## Conclusions

The “NAE/NAPE pathway,” including the enzymes synthetizing and degrading NAEs, their precursors and their receptors, represents a crucial regulatory pathway for many physiological functions and may become the target for the development of novel therapeutic drugs for many pathological conditions. In this review, we outlined recent progress in the studies of the mechanisms mediating the anorexic effects of OEA and NAPEs. Among NAEs, OEA may represent the optimal candidate for the development of anti-obesity treatments and NAPEs continue to be an intriguing and interesting group of phospholipids, both as NAE precursors and in their own right.

### Conflict of Interest Statement

The associate editor, Cesare Mancuso declares that, despite having collaborated with the author Silvana Gaetani, the review process was handled objectively and no conflict of interest exists. The authors declare that the research was conducted in the absence of any commercial or financial relationships that could be construed as a potential conflict of interest.
